# Assessment of Lethal, Sublethal, and Transgenerational Effects of *Beauveria bassiana* on the Demography of *Aedes albopictus* (Culicidae: Diptera)

**DOI:** 10.3390/insects11030178

**Published:** 2020-03-11

**Authors:** Rana Fartab Shoukat, Junaid Zafar, Muhammad Shakeel, Yuxin Zhang, Shoaib Freed, Xiaoxia Xu, Fengliang Jin

**Affiliations:** 1 Key Laboratory of Bio-Pesticide Innovation and Application of Guangdong Province, College of Agriculture, South China Agricultural University, Guangzhou 510642, China; ranafartab@gmail.com (R.F.S.); jz_jaam@yahoo.com (J.Z.); faizaneabiwaqas@scau.edu.cn (M.S.); listzhang@163.com (Y.Z.); xuxiaoxia111@scau.edu.cn (X.X.); 2 Laboratory of Insect Microbiology and Biotechnology, Department of Entomology, Faculty of Agricultural Sciences and Technology, Bahauddin Zakariya University, Multan 66000, Pakistan; sfareed@bzu.edu.pk

**Keywords:** Asian tiger, mosquito, demography, entomopathogenic fungi, *Beauveria bassiana*

## Abstract

Dengue fever is one of the most rapidly spreading arthropod-borne diseases. Diurnal vectorial properties of *Aedes albopictus* contribute to the dispersion of the dengue viruses. Frequent and injudicious use of synthetic insecticides led to the evolution of resistant phenotypes in *Ae. albopictus* which necessitates the search for an alternative of current control strategies. Developing a long-lasting and environmentally safe tactic based on knowledge of ecology and population dynamics of *Ae. albopictus* is critical. Therefore, with a view towards biological control and ecology, the effect of entomopathogenic fungi (EPF) *Beauveria bassiana* on filial and first filial generations of *Ae. albopictus* were studied. Investigations showed 87.5% adulticidal activity leading to altered fecundity and adult longevity in a filial generation. The lethal (LC_50_) and sublethal (LC_20_) concentrations of *B. bassiana* were applied to filial generation (F_0_) to study demographic parameters in the first filial generation (F_1_). Results showed reduced net reproductive rates (*R_o_)* intrinsic rate of increase (*r*)*,* and mean generation time (*T)* compared to uninfected controls. Prolonged larval and pupal duration were observed followed by reduced longevity of male and female adults. Fecundity in the first filial generation was significantly changed with the lethal and sublethal concentrations of *B. bassiana.* Thus, it is concluded that *B. bassiana* has the potential to play a vital role in integrated mosquito management strategies.

## 1. Introduction

Mosquito-borne diseases have been a primary concern for the human population for a long time. Due to *Aedes albopictus,* more than 100 nations in tropical and subtropical regions around the world are confronting the dangers of dengue fever, yellow fever, and chikungunya [1]. Diurnal and repeated blood-feeding behavior of *Ae. albopictus* makes it more dangerous than other mosquito species, it also facilitate the rapid transmission of diseases [2]. Moreover, past injudicious use of synthetic insecticides against larvae and adults of mosquito led to the evolution of resistant phenotypes [3], environmental contamination, and human health risks. Specifically, indoor use of synthetic insecticides against medically-important pests like mosquito negatively impact human health [4,5]. Non-target and broad spectrum insecticides negatively impact biodiversity [6,7,8] and life cycles of non-target insects [9,10,11]. All these issues contribute for the need of research for alternative control methods which would be long-lasting and safe for the environment and humans. As biological control agents, entomopathogenic fungi (EPF) are cosmopolitan and can be used safely against insect pests [12,13,14,15,16,17,18,19,20,21]. In recent studies, EPF have also shown promising adverse effects on different mosquito species [22,23,24].

EPF can be used more efficiently if the impacts of the life cycle of *Ae. albopictus* are better known. Population dynamics and population ecology of insects play a vital role in the development of long-lasting control with EPF [22,25,26]. Life table analysis of *Ae. albopictus* can help to determine the best time and stage upon which EPF should be applied for management [27,28].

Life table studies for *Ae. albopictus* have been conducted with a focus on immature stages but the adult populations (male especially) are overlooked [29]. Life table analysis without the inclusion of the male population cannot predict valid demographic parameters; hence age stage two-sex life tables are used [30,31,32]. The age stage two-sex life table, developed by Chi [33] subsequently and mathematically proven [34], can differentiate the stage and male populations.

The current study was planned with *Ae. albopictus*, and assessments of *Beauveria bassiana* isolated from two different sources were done. The best isolate was used to examine transgenerational effects on demographic parameters of filial offspring and first generations offspring by utilizing the age stage two-sex life table, which will guide us towards the effective use of *B. bassiana* at the most impactful time and stage of *Ae. albopictus*. This will contribute to development of a useful, and eco-friendly tool for integrated pest management of this urban pest.

## 2. Material and Method

### 2.1. Rearing of Ae. albopictus

*Ae. albopictus* (Foshan strain) eggs were collected from Guangdong Center for Disease Control and Prevention Guangzhou, China (2017). Eggs were brought on disinfected media (jars cleaned with ethanol 90%, air dried 30 mints) to the Laboratory of Bio-Pesticide Innovation and Application of Guangdong Province, College of Agriculture, South China Agricultural University, Guangzhou, China. Filter papers containing the eggs were placed in glass beakers (200 mL) containing water for hatching under controlled (Temperature: 28 ± 2 °C, Relative Humidity:64 ± 5% and photoperiod 11:13 h light:dark) laboratory conditions (pathogen-free environment) [35]. Two-day old post-hatching larvae were transferred in 1000 mL glass jars (15 × 10 cm). Well-ground fish food (Godzilla, CST945) was provided to larvae as food [36]. Pupae were collected and placed in a separate glass beaker (200 mL) which was placed in white cloth cage (30 × 30 cm) for adult emergence [22]. Male and female adults were provided a 10% sugar solution (w/v) while egg-laying female adults were blood-fed on mice with ethical approval (SCAU-AEC-2010-04-16) by the Guangdong Province Administration Office of Laboratory Animals. Conical shaped wet filter paper fitted in a 200 mL glass beaker (filled with distilled water) was used as an oviposition site.

### 2.2. Fungi Culture

Two soil extracted isolates of *B. bassiana* (Bb-01 and Bb-10), were obtained from the Laboratory of Insect Microbiology and Biotechnology, Bahauddin Zakariya University, Multan, Pakistan [24,37]. Bb-01 was isolated from multan Punjab Pakistan (30°05′11.65″N 71°39′15.65″E) while Bb-10 was isolated from soil of Mansehra, Khyber Pakhtunkhwa, Pakistan (34°20′2″N 73°12′5″E).

Isolates were passaged multiple times to prevent aging [38]. A two-week-old potato dextrose agar grown monoconidial culture maintained at 25 °C was used in suspension formation. For stock solutions, disinfected spatulas were used to harvest the conidia in 0.05% Tween-80 (Sigma-P1754) diluted in distilled water [39]. Stock solutions were stored at 4 °C for further use [40,41]. Serial dilutions were made to provide the desired concentrations from the stock solution.

### 2.3. Bioassay

#### 2.3.1. Screening of *Beauveria bassiana* Isolates

Two isolates of *B. bassiana* (Bb-01 and Bb-10) were screened out against the adult (2 days old males and females) of *Ae. albopictus*. Five concentration (3 × 10⁸, 3 × 10^7^, 3 × 10^6^,3 × 10^5^, 3 × 10^4^ spores/mL) were prepared for both isolates, whereas 0.05% Tween-80 (Sigma-P1754) diluted in distilled water was taken as tween control and distilled water was a general control [39]. Plastic jars (10 × 10 × 10 cm) were sprayed with fungal concentrations (10 mL) and air-dried for two hours. Ten adults were added to the jar of each concentration of every isolate. The whole experiment was repeated four times. Sugar solution (10%) was provided to male and female while the additional blood meal was provided to females for egg-laying. All experiments were conducted under controlled laboratory conditions as described above. Data regarding adult mortality for 7 days (24 h interval) [12,13,22]. Adults showing no movement were considered dead.

#### 2.3.2. Selection of *Beauveria bassiana* Isolate

The LC_50_ was calculated for both isolates of *B. bassiana* (POLO-PC software). The fungal isolate with the lowest LC_50_ was chosen for further experimentation with the age stage two-sex life table.

#### 2.3.3. Assay of Blood-Fed Females

A selected isolate of *B. bassiana* was again tested on blood-fed and non-blood-fed females, five concentrations (3 × 10⁸, 3 × 10^7^, 3 × 10^6^, 3 × 10^5^, 3 × 10^4^ spores/mL) were prepared and applied with the same methodology as described above. Females used in experimentation were 2 days old (24 h starved) and concentrations were applied 8 h post blood-feeding.

#### 2.3.4. Validation of Lethal (LC_50_) and Sublethal (LC_20_) Concentrations

Lethal and sublethal concentrations (LC_50_, LC_20_) for the life table studies were practically evaluated against adults of *Ae. albopictus*. For experimentation jars (10 adult/replication) sprayed with desired concentrations were used with the same procedure as described above.

#### 2.3.5. Influence of *Beauveria bassiana* on Longevity and Fecundity of Filial Generation (F_0_)

A total of 1000 newly emerged adults (1:1) were selected for bioassays. Males and females (250 each) were separated with an electric aspirator and subjected to Lethal (LC_50_) and sublethal (LC_20_) concentrations separately, in treated jar assays (500 adults, 1:1) water diluted 0.05% Tween-80 (Sigma-P1754) was used as control. Mortality was observed every 24 h for 7 days, after the seven days of treatment the remaining adults of filial generation (F_0_) were copulated (1pair/cage) in plastic cages covered with a white cloth. Blood meals were provided every four days until the death of females (Scholte et al. 2006). Glass beakers with wet filter paper were used as ovipositional sites. Fecundity and longevity were observed until the death of every individual of the filial generation [42].

#### 2.3.6. Transgenerational Effect of *Beauveria bassiana* on First Filial Generation (F1)

Eggs laid by filial generations, 100 eggs were collected from each group (LC_50_, LC_20_, and control). Eggs were placed individually on disinfected plastic trays (150 mL distilled water) without fungal exposure in pathogen-free environment. Powered fish food (Godzilla, CST945) was individually given to larvae as food. The transgenerational effects were studied from larva to adult stage. Data were recorded every 12 h until the death of all individuals. On the emergence of adults, individuals of the first filial generation were paired and shifted in cages for seeking the data regarding longevity and fecundity.

### 2.4. Statistical Analysis

The calculation of lethal and sublethal concentrations was conducted by using POLO-PC software [43]. Mortality data were analyzed by one-way ANOVA. Means were separated by using Tukey’s HSD test in Minitab 16 software at a 5% level of significance. Life table parameters like development, fecundity and, longevity were obtained by using the age-stage two-sex life table [33,44,45,46]. The bootstrap technique (*n* = 100,000) was used for the calculation of standard errors for life table parameters [47]. The program (TWO SEX-MS Chart) for age-stage two-sex life table analysis was designed in visual basics for the window operating system and can be obtained from the following link [33,46] (http://140.120.197.173/Ecology/prod02.html) (Chung Hsing University).

In age stage two-sex life table net reproductive rate (*R*_0_), which shows a total number of offspring by an individual throughout its life was calculated via the equation

(1) $$R_{0}=\overset{\infty }{\underset{x=0}{\sum }}l_{x}m_{x}$$

while *l_x_*, the probability of a newly laid egg surviving to age x can be calculated as

(2) $$l_{x}=\overset{k}{\underset{j=1}{\sum }}s_{xj}$$

*m_x_* is mean fecundity of individuals at age x can be obtained from the following equation

(3) $$m_{x}=\frac{\sum_{j=1}^{k}s_{xj}f_{xj}}{\sum_{j=1}^{k}s_{xj}}$$

The intrinsic rate of increase (r) was evaluated utilizing the iterative bisection strategy and adjusted with the Euler–Lotka condition with the age-indexed (Goodman 1982).

(4) $$\overset{\infty }{\underset{x=0}{\sum }}e^{-r\left(x+1\right)}l_{x}m_{x}=1$$

The finite rate was calculated as

(5) $$\lambda =e^{r}$$

Length of time that a population needs to increase to *R*_0_-fold of its population size at the stable age-stage distribution is called as mean generational time, and is calculated as

(6) $$T=1nR_{0}|r$$

*e_xj_*, the length of time that an individual of age *x* and stage *j* is expected to live could be obtained from the equation below [44]

(7) $$e_{xj}=\overset{\infty }{\underset{i=x}{\sum }}\overset{\beta }{\underset{y=j}{\sum }}S_{iy}^{\prime }$$

The reproductive value (*v_xj_*) was calculated according to [48,49] and was calculated as

(8) $$V_{xj}=\frac{e^{r\left(x+1\right)}}{s_{xj}}\overset{\infty }{\underset{i=x}{\sum }}e^{-r\left(i+1\right)}\overset{\beta }{\underset{y=j}{\sum }}S_{iy}^{\prime }f_{iy}$$

## 3. Results

### 3.1. Screening of Beauveria bassiana Isolate

**Figure 1:**
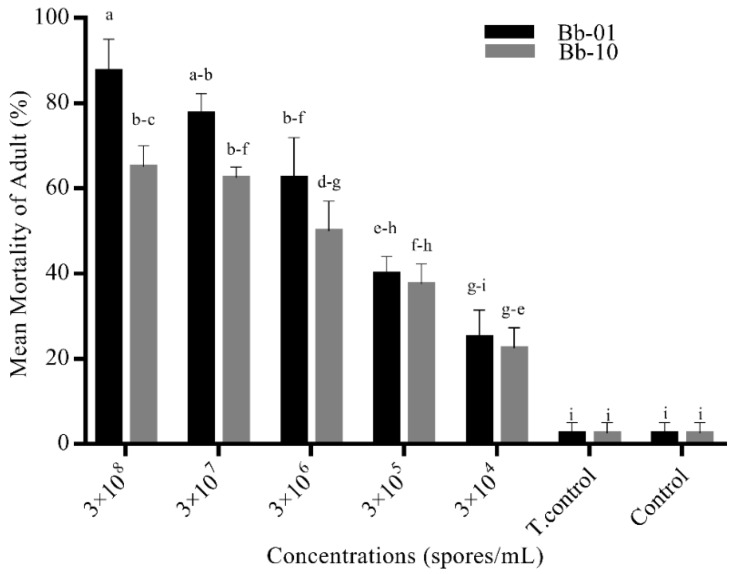
Adulticidal activity of *B. bassiana* (isolates). Green bars represent the mortality of adult *Ae. albopictus*, after exposure to different concentrations of *B. bassiana* first isolate (Bb-01). Red bars represent the mortality of adult *Ae. albopictus* to different concentrations of the second isolate (Bb-10) from *B. bassiana*. Error bars show 95% confidence intervals (CI). Different letters indicate significant differences at *p* < 0.05.

Both isolates of *B. bassiana* (Bb-01, Bb-10) revealed a direct relationship between concentration and mortality (Figure 1). At the maximum concentration of 3 × 10⁸ (spores/mL), Bb-01 exhibited the highest adulticidal activity (87.5 ± 0.47) followed by Bb-10 (65 ± 0.5). Mortality in both control treatments was low and lowered then all concentrations of both isolates (*p* < 0.05, DF = 4) dead specimens were put in humid chamber for conidal growth (supplementary material)

### 3.2. Selection of Beauveria bassiana Isolate

Lethal (LC_50_) and sublethal (LC_20_) doses for both isolates (Bb-01, Bb-10) of *B. bassiana* were calculated from pre-experimentation data (Table 1). The isolate with the lowest LC_50_ was selected for further studies; hence *B. bassiana* isolate Bb-01 met the desired criteria.

### 3.3. Assay of Blood-Fed Females

**Figure 2:**
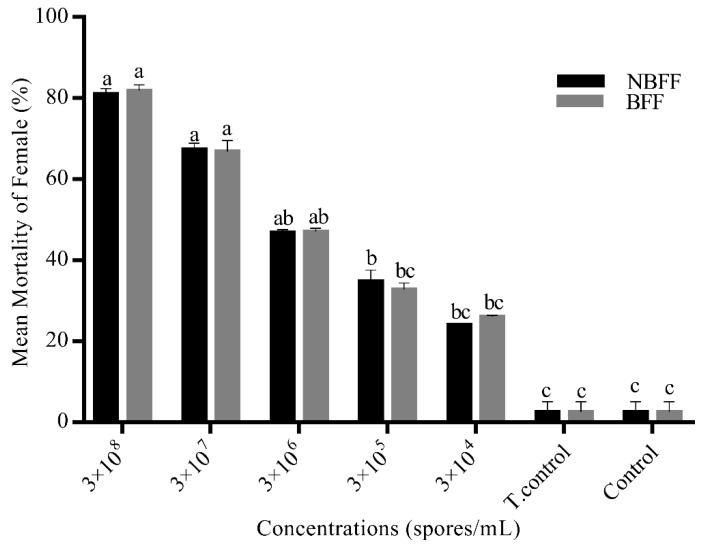
Feeding assay of *B. bassiana* (isolates) against *Ae. albopictus* (females). Black bars represent the mortality of non-blood-fed females on different concentrations of selected *B. bassiana* isolate (Bb-01), while grey bars represent the mortality of blood-fed females. Error bars show 95% confidence intervals (CI). Different letters indicate significant differences at *p* < 0.05.

Blood-fed and non-blood fed females of *Ae. albopictus* were exposed to five concentrations of *B. bassiana* (Bb-01) by contact assay in the jar. Non-significant results were seen (Figure 2) between blood-fed and non-blood fed females. Blood meal did not affect the adulticidal activity of *B. bassiana* (Bb-01).

### 3.4. Validation of Lethal (LC_50_) and Sublethal (LC_20_) Concentrations

Calculated lethal (LC_50_) and sublethal (LC_20_) concentrations of *B. bassiana* (Bb-01) were experimentally validated, lethal concentration LC_50_ showed 51.54 ± 0.98% adult mortality followed by sub-lethal concentration LC_20_ (23.11 ± 1.11).

### 3.5. Influence of Beauveria bassiana on Longevity and Fecundity of Filial Generation (F_0_)

After the treatment of Lethal (LC_50_) and sublethal (LC_20_) concentrations of *B. bassiana* (Bb-01), the effect of longevity and fecundity of *Ae. albopictus* (filial generation, F_0_) were seen (Table 2). For the filial generation exposed to LC_50_ and LC_20_ of Bb-01, male longevity was reduced to 21.33 and 26.09 days respectively, while maximum male longevity was seen in controls (29.91 ± 1.20). Female longevity also showed a similar tread to male longevity (days), where LC_50_ of Bb-01 presented the lowest longevity (22.22 ± 1.21) followed by LC_20_ of Bb-01 (27.65 ± 1.77) as compared to the control (30.07 ± 0.41). A significant reduction in fecundity (eggs/female) was also observed. Exposure to LC_50_ of Bb-01 results in the fewest number of eggs (189.31 ± 8.11) followed by the LC_20_ exposure (230.47 ± 9.32) and controls (357.33 ± 9.30).

### 3.6. Effect of Beauveria bassiana on First Filial Generation (F1)

For evaluation of transgenerational changes in the first filial generation of *Ae. albopictus,* an age stage two-sex life table was used. Basic parameters of life table such as developmental time, longevity, and fecundity are presented in Table 3. Egg hatching of 100% was observed with both control and sub-lethal (LC_20_) concentration of *B. bassiana,* while 95% of egg hatching was observed after treatment of lethal (LC_50_) concentration of Bb-01. An opposite trend was observed in egg duration, where control (2.00 ± 0.01 days) and sublethal concentration (LC_20_) of *B. bassiana* (2.00 ± 0.01) showed prolongs the egg hatching duration, while early egg hatching (days) was seen in lethal (LC_50_) concentration (1.95 ± 0.54) of *B. bassiana*. Significantly different total larval duration was reported, LC_50_ of *B. bassiana* showed maximum (8.38 ± 0.16) development time (days) follow by LC_20_ (7.97 ± 0.01), while minimum developmental time (7.89 ± 0.03) was seen in the control group. In pupal duration (days), LC_50_ of *B. bassiana* showed minimum pupal duration (2.55 ± 0.59) then LC_20_ (3.00 ± 0.59) and control (3.00 ± 0.11). A significant reduction was seen in the life span of male *Ae. albopictus*, an increase in the concentration of *B. bassiana* had an inverse effect on the reduction of the male life span. Male adults of *Ae. albopictus* showed minimum life span (27 days) on LC_50_ of *B. bassiana* then LC_20_ (30.10 days) and control (30.00 days). A similar trend was observed in the female life span. Fecundity had an inverse relation with lethal and sublethal concentrations of Bb-01, females treated with LC_50_ of *B. bassiana* laid a minimum mean number of eggs (320/female), while females from LC_20_ of *B. bassiana* laid more eggs (349/female) then LC_50_. The maximum mean number of eggs was observed in control (380/female).

Population parameters calculated with the help of the age stage two-sex life table are shown in Table 4. The intrinsic rate of increase (*r*) was inversely related to concentration, which varied from 0.27 to 0.25 and 0.23 in the control, LC_20_, and LC_50_ respectively. Mean finite rate of increase (λ) had a significant difference (per day) between control (1.32 ± 0.01), LC_20_ (1.29 ± 0.02) and LC_50_ (1.26 ± 0.06). The net reproduction rate (*R_0_*) (offspring/individual) was high in control (133.0 ± 16.21) and then gradually decrease significantly in LC_20_ (89.16 ± 9.31) and LC_50_ (50.62 ± 8.31). Significant differences were also observed between mean generation times (*T*), 17.83 days for control and 17.80 and 17.09 days in LC_20_ and LC_50_ respectively (*p* < 0.05).

**Figure 3:**
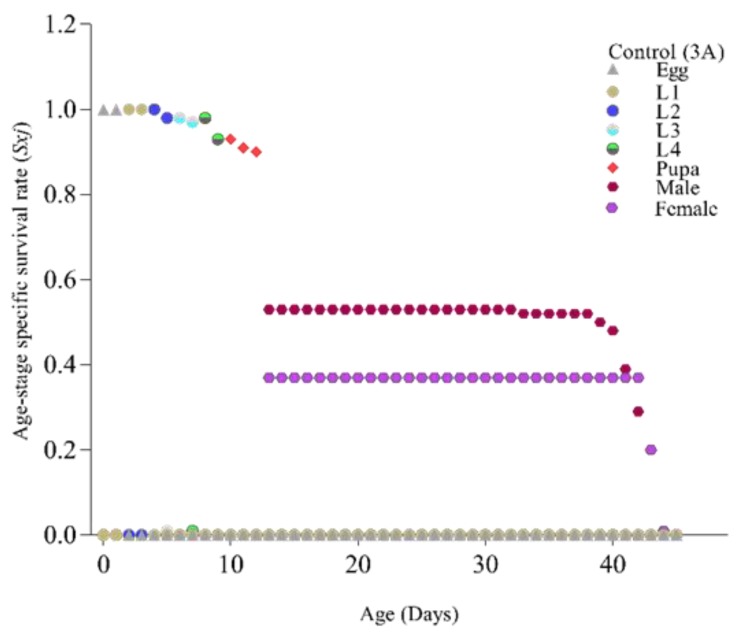

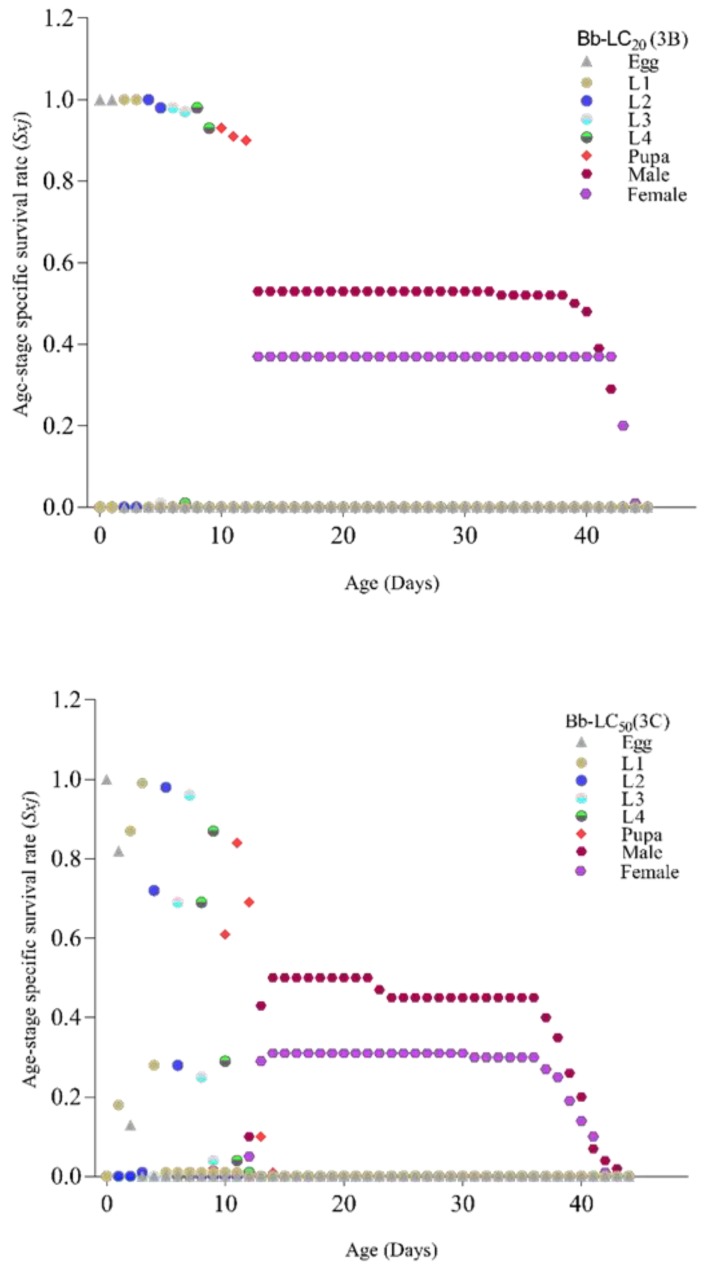
Age-stage specific survival rate (Sxj) of Ae. albopictus after treatment with *Beauveria bassiana*: **3A** (control), **3B** (LC20), **3C** (LC50). *Ae. albopictus* age-stage specific survival rate (Sxj) of the control group is shown as Figure 3A, while Figure 3B,C showed the age-stage specific survival rate (Sxj) after exposure to sub-lethal (LC20) and lethal (LC50) concentrations of a selected isolate of *B. bassiana* (Bb-01) respectively. Life stages are shown in distinctive colors.

**Figure 4:**
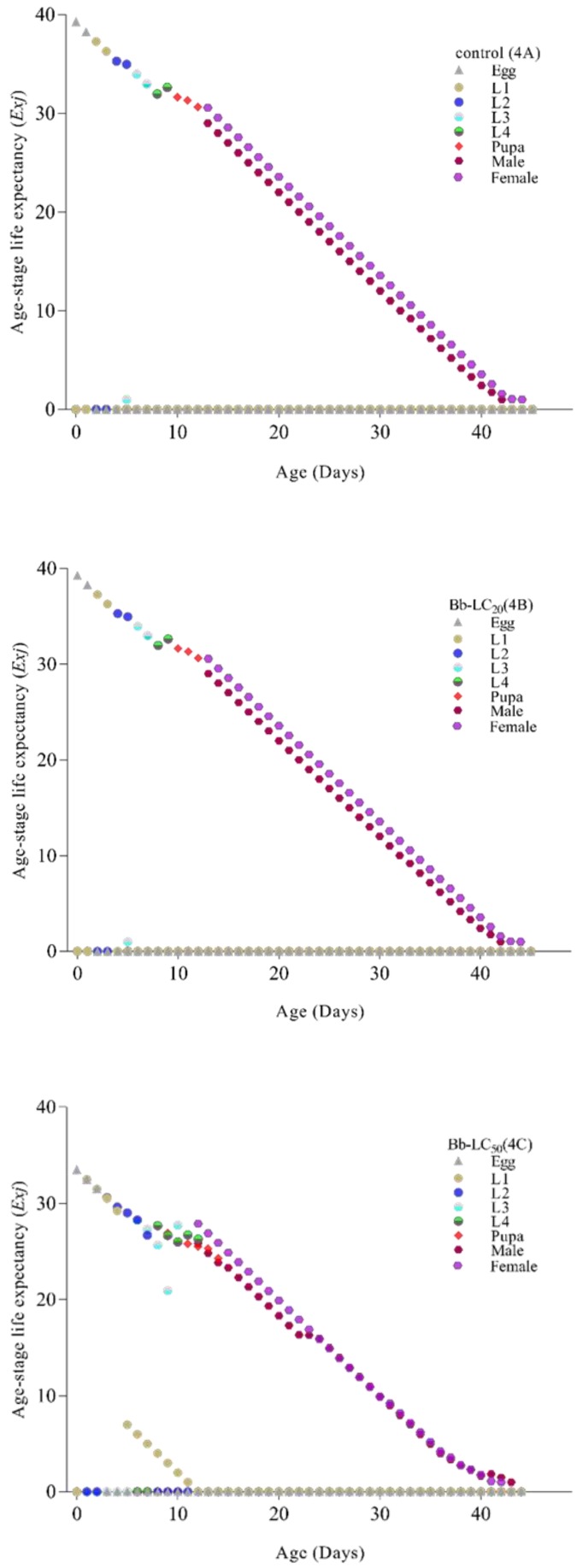
Age-stage life expectancy (Exj) of *Ae. albopictus* after treatment with *Beauveria bassiana*: **4A** (control), **4B** (LC20), **4C** (LC50). *Ae. albopictus* age-stage life expectancy (Exj) of the control group is shown as Figure 3A, while Figure 3B,C showed the age-stage life expectancy (Exj) after exposure to sub-lethal (LC20) and lethal (LC50) concentrations of a selected isolate of *B. bassiana* (Bb-01) respectively. Life stages are shown in distinctive colors.

The age stage survival rate (*Sxj*) signifies that in the first filial generation overall life span of *Ae. albopictus* in the control group was longer and was reduced after treatment of LC_20_ and LC_50_ of *B. bassiana* (Figure 3A–C, respectively). A similar trend was also observed in age-stage life expectancy (*Exj*) where treated group individuals had a lower life expectancy and overall life span (Figure 4A–C).

**Figure 5:**
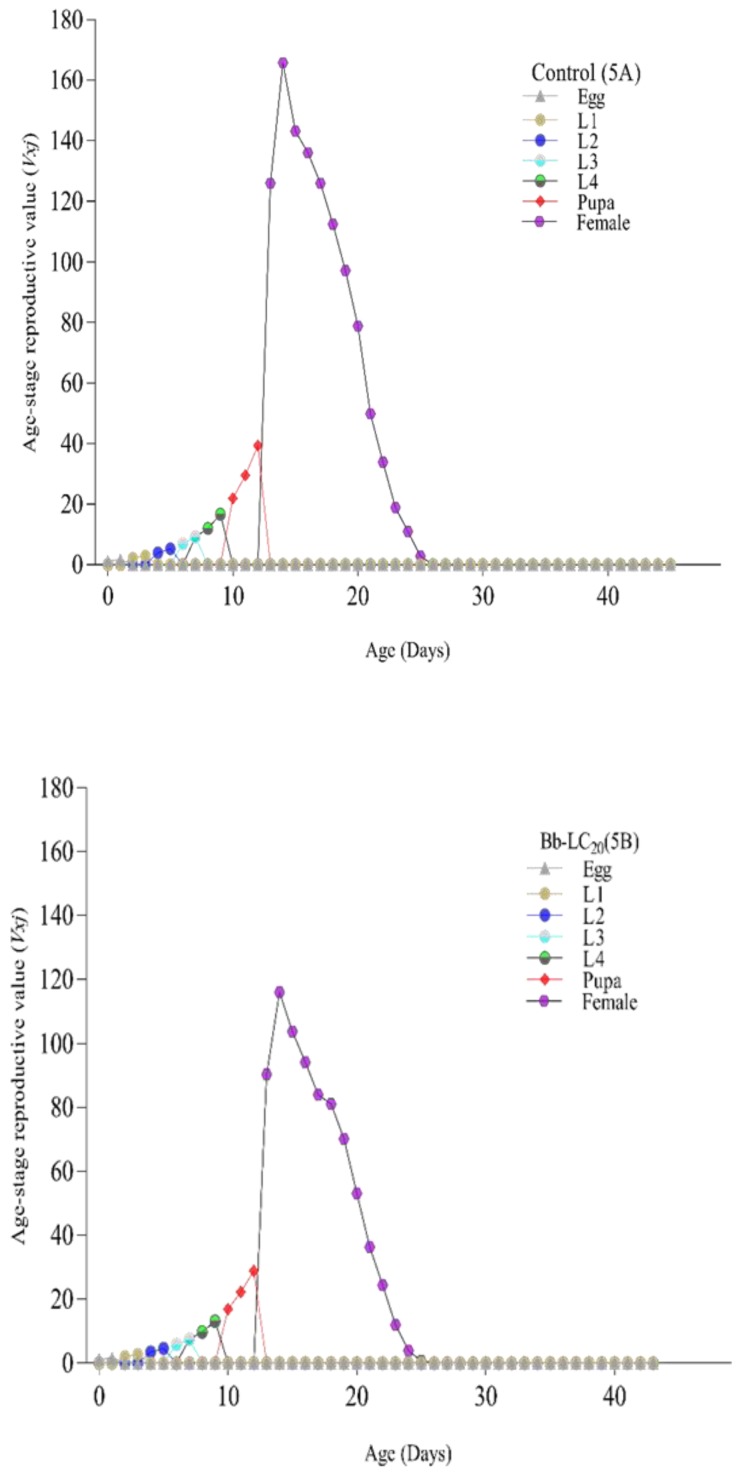

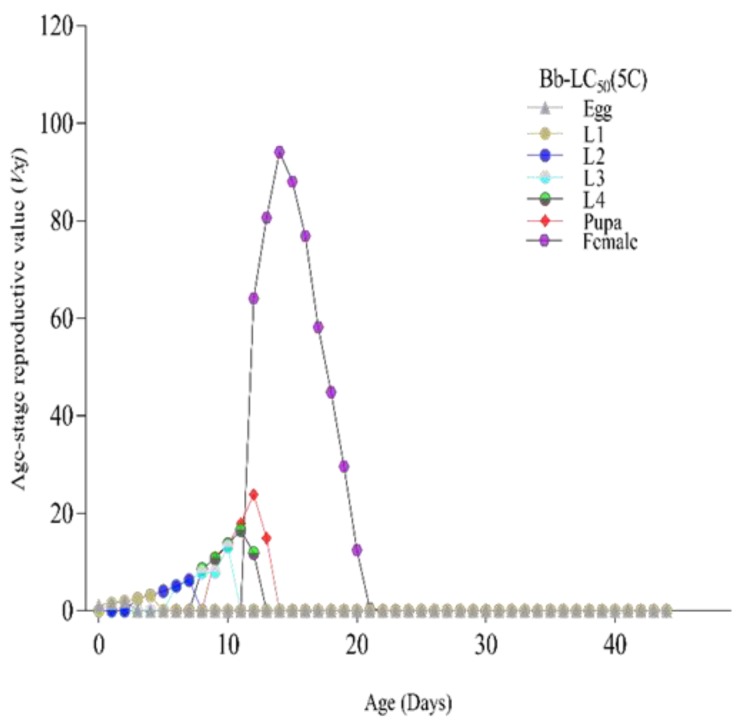
Age stage reproductive value (Vxj) of *Ae. albopictus* after treatment with *Beauveria bassiana*: **5A** (control), **5B** (LC20), and **5C** (LC50). *Ae. albopictus* age stage reproductive value (Vxj) of the control group is shown as Figure 3A, while Figure 3B,C showed the age stage reproductive value (Vxj) after exposure to sub-lethal (LC20) and lethal (LC50) concentrations of a selected isolate of *B. bassiana* (Bb-01) respectively. Life stages are shown in distinctive colors.

**Figure 6:**
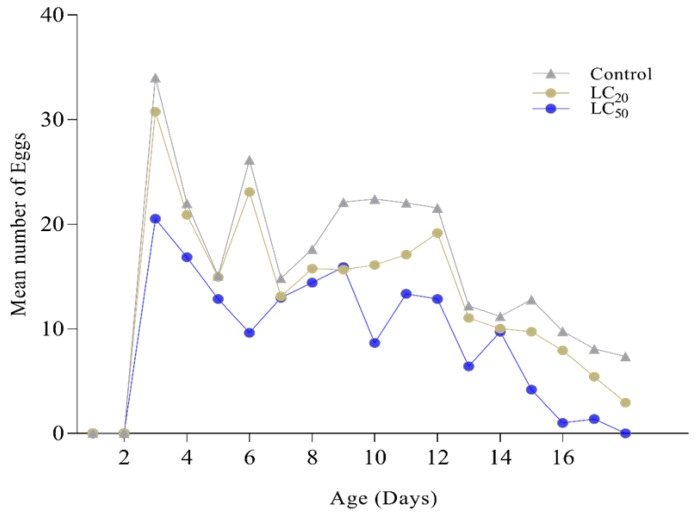
Daily mean number of eggs from *Ae. albopictus* after treatment with *Beauveria bassiana* at LC20 and LC50 doses. The figure is showing the daily reproduction rate of females, where the control group is represented with the gray color line while sub-lethal (LC20) and lethal (LC50) concentrations of *B. bassiana* selected isolate (Bb-01) are presented with yellow and blue colors lines respectively.

Age stage reproductive value (*Vxj*) shows the maximum reproductive value of a stage in life span, adult females from the control group showed the highest peak of reproductive value as compared to lethal and sublethal concentrations of *B. bassiana* (Figure 5). Daily reproduction (Figure 6) also showed a significant difference in the mean number of eggs (*p* < 0.05). Egg-laying started 2 days post blood meal; in the beginning, controlled females showed a maximum number of eggs followed by females of LC_20_ and LC_50_. With time reduction in a mean number of eggs was seen in treated females.

## 4. Discussion

The life table of tracheal arthropods (insects) is directly related to the control of insects. Increased knowledge of insect stages, survival, and reproduction, can lead us towards promising and long-lasting control of insect pests [36,50,51,52,53,54,55]. Two virulent isolates of *B. bassiana* (extracted from different soils) were screened against *Ae. albopictus* with both isolates showing noteworthy adulticidal activity. The isolate extracted from soil of (Bb-01) in Pakistan showed more virulence than the isolate from the soil of cold area (Bb-10) soil. Bb-01 provided 87.5% adult mortality which also goes in favor of previous studies where *Ae. albopictus* and *Culex* spp. showed 80–90% adult mortality after treatment of EPF [23,40,56,57]. Due to the least LC_50_ and high adulticidal activity, Bb-01 was selected for further studies to assess its transgenerational effects on the filial and first filial generation of *Ae. albopictus.*

Insect chitin made cuticle is the main line of defense against EPF. In the case of medical insects like mosquito, complete control (Mortality) via EPF is not so easy. While EPF causes mortality in two weeks, an essential aspect is the effect of EPF on life parameters of mosquito. *B. bassiana* produces secondary products like beauvericin, bassianin, bassianolide, beauverolides, beauveriolides, tenellin, oosporein, and oxalic acid which evade the humoral and immune system of the insect [58,59]. Hyphae of *B. bassiana* absorbs the sugar contents from the hemolymph of insect which contributes towards insect weakness and disrupts various biological parameters of insect pests [60,61]. The current study strengthens this argument because egg hatching, larval duration, pupal duration, adult longevity, and fecundity in a filial and first filial generation were affected significantly. Lower egg hatching was observed due to the treatment of lethal concentration (LC_50_) from Bb-01 which goes in favor of previous studies in *Aedes* spp and *S. litura* (Fabricius 1775) where EPF causes a reduction in egg hatching [62,63,64].

EPF penetrates the cuticle and affects the fat body of the insect depleting energy resource which directly affects the insect stadium [65,66,67], which resulted in prolonged larval and pupal duration. A similar trend was observed in the current study, where the lethal and sublethal concentration of Bb-01 showed long larval and pupal duration. Prolonged larval and pupal duration has also been reported in past studies of *Musca domestica* (Linnaeus 1758) and *Culex* spp. [22,23,68].

In epidemiological models for diseases of *Ae. albopictus*, adult control is the most important thing [69,70,71]. Short longevity of *Ae. albopictus* adults will help in the control of vector-borne diseases [72]. Our findings showed reduced male and female longevity in filial and first filial generation due to the treatment of lethal and sublethal concentrations of Bb-01, previously *Ae. albopictus* and *Ae. aegypti* had also shown a significantly short life span after infection of EPF [40,41,69]. *Anopheles* spp. also showed low adult survival after the treatment of EPF [69,73,74,75,76]. A similar trend was observed in *Rhynchophorus ferrugineus* (Olivier) and *Trogoderma granarium*, where 20-30% reduced longevity was observed after treatment of EPF [77,78].

The infection of EPF fluctuates the body temperature of females, resulting in the loss of appetite, which led to reduced fecundity [79]. In current studies, the concentration of Bb-01 was inversely proportional to the mean number of eggs from females of *Ae. albopictus*. In past studies reduction in blood-feeding [80], retardation in fecundity has been significantly reported due to infection of EPF [41]. *Musca domestica* and *S. litura* showed less mean number of eggs after the treatment of EPF [18,68]. *B. bassiana* showed a significant effect on all demographic parameters of *Ae. albopictus* in a filial and first filial generation.

## 5. Conclusions

This study is first of his kind and provides basic yet important time-specific and age-specific information for a better understanding of *Ae. albopictus* population dynamics under the influence of *B. bassiana*. From our investigation, the conclusion can be drawn that *B. bassiana* has lasting effects on the developmental parameters of *Ae. albopictus*. Mainly the impact of the Bb-01 on adult longevity and female fecundity was significantly affected. Considering the impact of *B. bassiana* on transgenerational parameters of filial and first filial generation, it can be integrated with effective dengue vector control strategies.

## Figures and Tables

**Table 1:** Lethal and sublethal doses of *Beauveria bassiana* isolates (spores/mL).

Isolates	LC_50_	LC_20_	Slop ± SE	χ^2^	*p*-Value	*df*
Bb-01	3.0 × 10^6^	2.1 × 10^3^	0.296 + 0.042	1.023	0.796	4
Bb-10	1.4 × 10^7^	3.2 × 10^4^	0.515 + 0.048	16.477	0.001	4

**Table 2:** Influence of *Beauveria bassiana* on adult longevity and fecundity of filial generation (F_0_).

Parameters	Means ± SE		
	Control	*B. Bassiana* (LC_20_)	*B. Bassiana* (LC_50_)
Adult mortality (days)	1.9 ± 0.17 ^c^	23.11 ± 1.11 ^b^	51.54 ± 0.98 ^a^
Male longevity (*n* = 50)	29.91 ± 1.20 ^a^	26.09 ± 2.01 ^b^	21.33 ± 3.21 ^c^
Female longevity (*n* = 50)	30.07 ± 0.41 ^a^	27.65 ± 1.77^b^	22.22 ± 1.21^c^
Fecundity (1/50)	357.33 ± 9.30^a^	230.47 ± 9.32^b^	189.31 ± 8.11^c^

Male longevity, Female longevity = Days. Means in the same row followed by the same letter are not significantly different (*p* > 0.05).

**Table 3:** Effect of *Beauveria bassiana* on first filial generation (F1).

Parameters	Control	LC_20_ Treated	LC_50_ Treated
	Means ± SE	Means ± SE	Means ± SE
Percent hatching	100 ± 0.00 ^a^	100 ± 0.00 ^a^	95 ± 2.11 ^b^
Egg duration	2.00 ± 0.01 ^a^	2.00 ± 0.00 ^a^	1.95 ± 0.54 ^b^
L1	2.00 ± 0.02 ^b^	1.99 ± 0.00 ^b^	2.31 ± 0.71 ^a^
L2	1.99 ± 0.11 ^b^	1.99 ± 0.10 ^b^	2.02 ± 0.61 ^a^
L3	1.99 ± 0.10 ^a^	1.99 ± 0.10 ^a^	1.99 ± 0.54 ^a^
L4	2.00 ± 0.02 ^b^	2.00 ± 0.00 ^b^	2.06 ± 0.59 ^a^
Total Larval duration	7.89 ± 0.03 ^c^	7.97 ± 0.01 ^b^	8.38 ± 0.16 ^a^
Pupal duration	3.00 ± 0.11 ^a^	3.00 ± 0.59 ^a^	2.55 ± 0.59 ^b^
Pre-oviposition period	13.00 ± 0.00 ^a^	13.01 ± 0.00 ^a^	12.95 ± 0.59 ^b^
Female longevity	32.95 ± 0.53 ^a^	32.00 ± 2.79 ^a^	29.00 ± 2.81 ^b^
Male longevity	30.00 ± 1.67 ^a^	30.10 ± 1.53 ^a^	27.00 ± 3.45 ^b^
Fecundity	380.27 ± 11.12 ^a^	349.87 ± 7.31 ^b^	320.00 ± 5.42 ^c^

L1 = 1st instar larva; L2 = 2nd instar larva; L3 = 3rd instar larva; L4 = 4th instar larva: Except for fecundity (eggs/female), units are days. Means in the same row followed by the same letter are not significantly different (*p* > 0.05).

**Table 4:** Population parameters of *Ae. albopictus* after treatment with *Beauveria bassiana*.

Parameters	Control	LC_20_ Treated	LC_50_ Treated
	Means ± SE	Means ± SE	Means ± SE
Intrinsic rate of increase (*r*)	0.2744 ± 0.007 ^a^	0.2506 ± 0.002 ^b^	0.2295 ± 0.005 ^c^
Net reproduction rate (*R_o_*)	133.0 ± 16.21 ^a^	89.16 ± 9.31 ^b^	50.62 ± 8.31 ^c^
Mean length of a generation (*T*)	17.83 ± 0.12 ^a^	17.80 ± 0.16 ^a^	17.09 ± 0.09 ^c^
Finite rate of increase (λ)	1.316 ± 0.01 ^a^	1.285 ± 0.02 ^b^	1.258 ± 0.06 ^c^
Birth rate (at SASD)	0.319 ± 0.12 ^a^	0.2883 ± 0.21 ^b^	0.264 ± 0.21 ^c^
Survival rate (at SASD)	0.997 ± 0.02 ^a^	0.997 ± 0.03 ^a^	0.995 ± 0.01 ^b^
Death rate (at SASD)	3.206 ± 1.03 ^c^	3.491 ± 1.04 ^b^	5.499 ± 1.07 ^a^

*r* = Intrinsic rate of increase (per days); *Ro* = Net reproduction rate (offspring/individual); *T* = Mean length of a generation (days); λ= Finite rate of increase (per days); SASD = Stable age-stage distribution. Means in the same row, followed by the same letter are not significantly different (*p* > 0.05).

## Supplementary Materials

The following are available online at https://www.mdpi.com/2075-4450/11/3/178/s1, Figure S1. Fungal growth of Beauveria bassiana isolate Bb-01 on adult Aedes albopictus (Culicidae: Diptera); Figure S2. Fungal growth of Beauveria bassiana isolate Bb-10 on adult Aedes albopictus (Culicidae: Diptera).
