# Supplementary material for: Assessment of Lethal, Sublethal, and Transgenerational Effects of Beauveria bassiana on the Demography of Aedes albopictus (Culicidae: Diptera)
Source: Insects. 2020 Mar 11;11(3):178. doi: 10.3390/insects11030178 (PMC7143237; doi:10.3390/insects11030178)
Supplement: Supplementary file 1 [file insects-11-00178-s001.pdf]

insects

MDPI

Article

Assessment of Lethal, Sublethal, and Transgenerational Effects of *Beauveria Bassiana* on the Demography of *Aedes Albopictus* (Culicidae: Diptera)

Rana Fartab Shoukat [1,†], Junaid Zafar [1,†], Muhammad Shakeel [1], Yuxin Zhang [1], Shoaib Freed [2], Xiaoxia Xu [1], and Fengliang Jin [1,*]

Supplementary

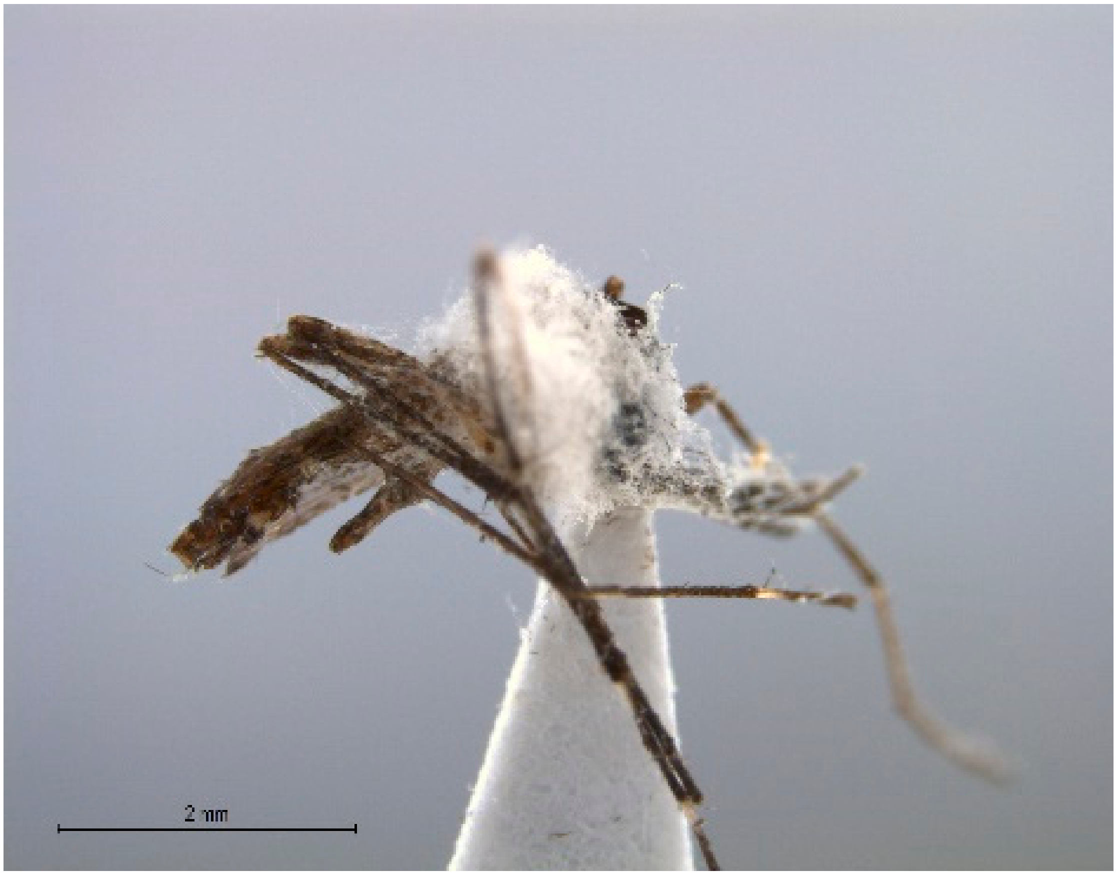

Figure S1. Fungal growth of *Beauveria bassiana* isolate Bb-01 on adult *Aedes albopictus* (Culicidae: Diptera). .

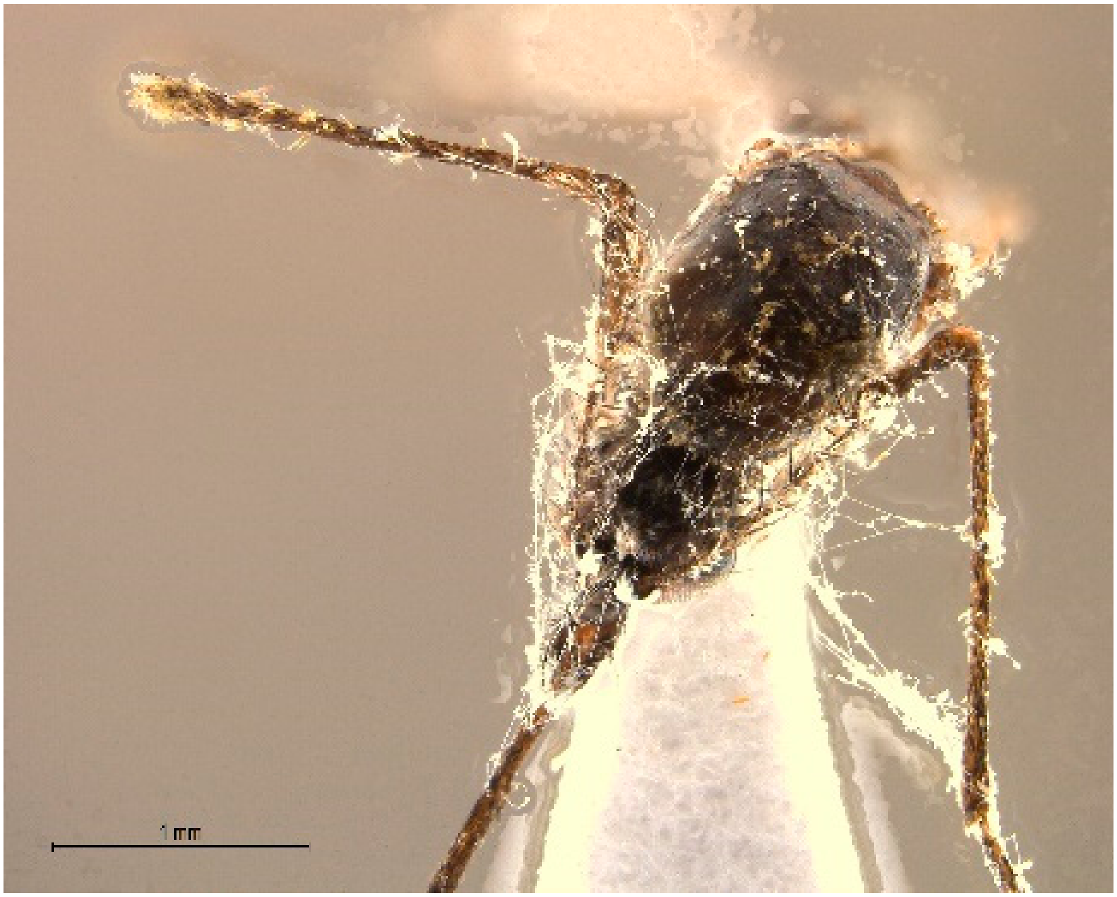

Figure S2. Fungal growth of *Beauveria bassiana* isolate Bb-10 on adult *Aedes albopictus* (Culicidae: Diptera).

© 2020 by the authors. Submitted for possible open access publication under the terms and conditions of the Creative Commons Attribution (CC BY) license (http://creativecommons.org/licenses/by/4.0/).
